# Youth experiences of transition from child mental health services to adult mental health services: a qualitative thematic synthesis

**DOI:** 10.1186/s12888-017-1538-1

**Published:** 2017-11-28

**Authors:** Kathleen L. Broad, Vijay K. Sandhu, Nadiya Sunderji, Alice Charach

**Affiliations:** 1 Huron Perth Healthcare Alliance School of Medicine, Stratford, ON Canada; 2 Faculty of Medicine, University of Toronto, Toronto, ON Canada; 3 Mental Health and Addictions Service, St. Michael’s Hospital, Toronto, ON Canada; 4 Li Ka Shing Knowledge Institute, Toronto, ON Canada; 5 Division of Adult Psychiatry and Health Systems, Department of Psychiatry, University of Toronto, Toronto, ON Canada; 6 Division of Child and Adolescent Psychiatry, Department of Psychiatry, University of Toronto, Toronto, ON Canada; 7 Program in Collaborative and Transitional Age Care, Department of Psychiatry, Hospital for Sick Children, Toronto, ON Canada

**Keywords:** Transition to adult care, Transitional programs, Health transition, Continuum of care, Adolescent, Young adult, Child adolescent psychiatry, Adolescent health services, Mental disorders, Mental health services

## Abstract

**Background:**

Adolescence and young adulthood is a vulnerable time during which young people experience many development milestones, as well as an increased incidence of mental illness. During this time, youth also transition between Child and Adolescent Mental Health Services (CAMHS) to Adult Mental Health Services (AMHS). This transition puts many youth at risk of disengagement from service use; however, our understanding of this transition from the perspective of youth is limited. This systematic review aims to provide a more comprehensive understanding of youth experiences of transition from CAMHS to AMHS, through a qualitative thematic synthesis of the extant literature in this area.

**Method:**

Published and unpublished literature was searched using keywords targeting three subject areas: Transition, Age and Mental Health. Studies were included if they qualitatively explored the perceptions and experiences of youth who received mental health services in both CAMHS and AMHS. There were no limitations on diagnosis or age of youth. Studies examining youth with chronic physical health conditions were excluded.

**Results:**

Eighteen studies, representing 14 datasets and the experiences of 253 unique service-users were included. Youth experiences of moving from CAMHS and AMHS are influenced by concurrent life transitions and their individual preferences regarding autonomy and independence. Youth identified preparation, flexible transition timing, individualized transition plans, and informational continuity as positive factors during transition. Youth also valued joint working and relational continuity between CAMHS and AMHS.

**Conclusions:**

Youth experience a dramatic culture shift between CAMHS and AMHS, which can be mitigated by individualized and flexible approaches to transition. Youth have valuable perspectives to guide the intelligent design of mental health services and their perspectives should be used to inform tools to evaluate and incorporate youth perspectives into transitional service improvement.

**Trial registration:**

Clinical Trial or Systematic Review Registry: PROSPERO International Prospective Register of Systematic Reviews CRD42014013799.

**Electronic supplementary material:**

The online version of this article (10.1186/s12888-017-1538-1) contains supplementary material, which is available to authorized users.

## Background

At least 75% of mental health problems and illness have onset in childhood, adolescence, or young adulthood [1]. However, this increased incidence of mental health conditions in youth corresponds to a weak point in mental health care provision [2]. The transition from Child and Adolescent Mental Health Services (CAMHS) to Adult Mental Health Services (AMHS) typically occurs between 18 and 21 years according to traditional age boundaries of service provision organizations, a period that overlaps important development milestones for emerging adults [3]. This is a vulnerable period [4] during which service users may disengage from utilizing mental health services at higher rates than other age cohorts [5, 6].

Many factors may contribute to youth disengagement, including disease-specific ambivalence or denial [7, 8] and the potential for mental illness and/or addictions to interfere with functioning and with acceptance of formal supports [9, 10]. It has also been postulated that differences between CAMHS and AMHS services may contribute to high disengagement rates [11]. However, overall factors contributing to disengagement, especially from the perspective of youth remain poorly understood.

Even when youth do receive care in AMHS, only 23% report finding the service helpful [12]. Gaps and suboptimal care during this vulnerable time have the potential for lasting functional impairment and development derailment [13, 14]. Age-specific outpatient programs have been shown to increase mental health service utilization, compared to standard adult outpatient programs [15]; however, they lack consistent evidence of effectiveness [16]. Given the vulnerability of this period and the unique needs of transition-aged youth, it is crucial to further understand youth experiences during the transition from CAMHS to AMHS.

Previous systematic reviews have shown that young people transitioning to adult health services experience concern over a loss of familiar surroundings and relationships [17] and want providers to be sensitive to their diverse needs [18]. Personal accounts from youth [19] and stakeholders [20, 21] have emphasized the need to directly involve young adults in the development of mental health services. Existing literature reviews of service transitions for youth with mental health concerns have identified gaps in the provision of transitional care [22], however, few of the included studies examined the experiences and perspectives of youth [23–25]. Thus, the youth voice in mental health planning and service delivery is under-represented, and their subjective experiences of transitioning from CMHS to AMHS are insufficiently understood.

Our primary aim is to understand and describe the subjective experiences of young people with mental health problems as they transition from the child and adolescent services to adult mental health services. This information will be helpful in planning services to address the needs of youth transitioning between service systems.

## Methods

In this systematic review, we examined youth experiences as they transition from CAMHS to AMHS. The scope was international, and focused on qualitative material because qualitative studies enable rich and open-ended exploration of subjective experiences. Such reviews have the advantage of providing a greater breadth and depth of understanding by accessing a larger number and diversity of service users accounts and a greater range of methodologies to elicit and analyze these accounts [26]. This review was designed as a thematic synthesis [27], a method that adapts approaches from both meta-ethnography and grounded theory and has been used in several systematic reviews examining people’s perspectives [26, 28]. Thematic synthesis allows our analysis to “stay close” to the expressed views of youth in the primary studies and retain particularities, while also allowing development of higher level themes occurring across multiple study populations to offer both cumulative and novel interpretations of the findings from primary studies as a whole [27]. Thus, by being interpretative and not merely aggregative, this type of synthesis can reduce uncertainty (e.g. in the case of recurrent themes across studies) and also enhance complexity (e.g. by highlighting differences and discrepancies) [29].

This review followed the “Enhancing Transparency in Reporting the Synthesis of Qualitative Research” (ENTREQ) guidelines [29] (See Additional file 1 for ENTREQ checklist) and consisted of (1) a systematic literature search for relevant qualitative and mixed methods research reports; (2) critical appraisal of included reports; and (3) inductive and iterative analysis of included reports. The protocol for this thematic synthesis was published a priori with PROSPERO International prospective register of systematic reviews (available online at http://www.crd.york.ac.uk/prospero/display_record.asp?ID=CRD42014013799; registration number CRD42014013799).

### Search strategy

We searched both published and unpublished (“grey”) literature. We identified published articles through systematic searches of the following electronic databases for academic journals from inception to October 2014: MEDLINE, EMBASE, PsycINFO, CINAHL, Social Services Abstracts, Applied Social Sciences Indexes and Abstracts (ASSIA); and of the following evidence-based medicine databases: The Cochrane database of systematic reviews, EBM Reviews, The Campbell Collaboration, and Centre for Reviews and Dissemination. A systematic search strategy was developed with the assistance of a librarian, and peer-reviewed by a second librarian. Keywords, their truncations and relevant database-specific subject headings and MeSH terms were used, targeting three subject areas: transition, age and mental health. For an example search strategy see Additional file 2.

We identified additional published literature through searches of reference lists of relevant articles (using Science Citation Index and hand searching) and forward citations of relevant articles (using Science Citation Index or Google Scholar). We identified unpublished literature through Google searches with the same keywords, and by contacting experts and key authors identified in the search of published literature.

### Inclusion and exclusion criteria

We included studies published in English that used a qualitative methodology to (1) Describe the perceptions and experiences of youth utilizing mental health services and (2) Explore their experiences of receiving services or care: (a) During the transition from CAMHS to AMHS setting or (b) In both the CAMHS and AMHS settings.

We included all studies examining young adults who have utilized mental health services, with no limitations on diagnosis, age, ethnicity or geographic locale. We excluded studies examining youth with exclusively chronic physical health conditions because young people utilizing mental health services have been much less studied and may experience unique challenges compared to young people with primarily physical disabilities [30].

All titles and abstracts were reviewed independently by two research team members (KB and VS) using DistillerSR, to organize the search, screen titles, and abstracts and extract data. Any differences were resolved by consensus amongst the two team members (KB and VS), and, if necessary, a third team member (NS). Inter-rater agreement for inclusion of studies was assessed using the chance-corrected Kappa statistic. Agreement for inclusion at the full text level ranged from Kappa scores of 86% to 95%.

### Quality assessment

We critically appraised all studies in duplicate (KB and VS) using the Critical Appraisal Skills Programme (CASP) Tool, which provides key criteria relevant to critically appraising qualitative research studies (e.g. appropriateness of research design, consideration of ethical issues, rigour of data analysis) [31]. Any differences were resolved by consensus amongst the two team members. All studies were included in final analysis.

### Data analysis

We conducted an initial content analysis of individual studies, followed by a thematic synthesis across all studies [27], focusing only on content representing the views of youth (typically, sections labelled “findings” or “results”).

Three of the authors (KB, VS and NS) independently read the text of three included studies and generated “codes”, and then met over multiple sessions to develop a consensus of codes and their meanings (i.e. a coding dictionary). We then continued to analyze three more studies independently, meeting regularly to triangulate perspectives and revise the coding dictionary by adding, merging, deleting, or modifying codes. Once the codes were not changing, the remainder of the included studies were coded by the first author (KB). KB then led the thematic analysis identifying over-arching themes emerging from the results of the studies as a whole and comparing and contrasting findings across studies and populations.

## Results

### Description of included studies

**Fig. 1:**
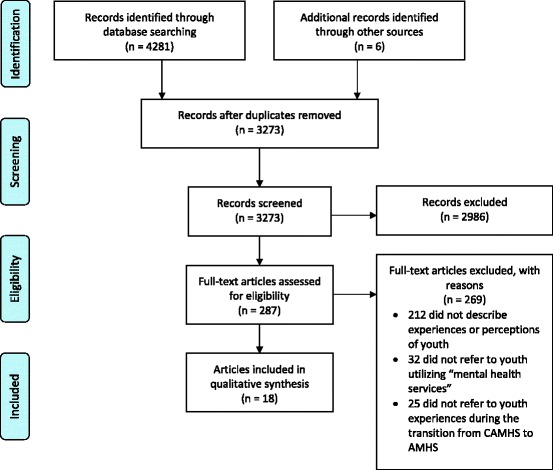
PRISMA diagram

We identified a total of 3273 abstracts, primarily through electronic databases with six articles identified through the other search strategies (see Fig. 1 for PRISMA diagram) [32]. Eighteen (18) articles, representing fourteen datasets met the inclusion criteria (see Table 1). Three articles reported findings from the TRACK Study: Singh [33], Singh et al. [6] and Hovish et al. [24] Two other article pairs also reported findings from the same datasets: (1) Munson et al. [34] and Munson et al. [35]; and (2) Lindgren et al. [36] and Lindgren [37]. Accounting for multiple reporting of datasets, across the 18 included articles, the experiences of 253 unique service-users were reported. Studies originated from the United Kingdom, United States, and Sweden. The age of participants ranged from 16 to 27 years old, and represented both young men and women, experiencing a range of diagnoses.

**Table 1:** Included Articles (18 articles representing 14 studies)

Study	N of Service-Users	Sample/Setting	Methods	Diagnosis	Age	Country	CASP Score (/10)
1. Beresford et al. [40]	4	Young people with high functioning autism on cusp of leaving school or who were young adults. Recruited from youth who responded to the family survey (done at selected local health trusts).	Interviews: topic guides with use of written chart as communication aid. Parents and professionals also interviewed. Thematic analysis.	High-functioning autism or Asperger’s syndrome.	18–19 years.	United Kingdom	8
2. Cheak-Zamora & Teti [42]	13	Convenience sampling from clinics seeing youth with ASD.	Semi-structured focus groups. Caregivers also interviewed. Thematic analysis.	Autism Spectrum Disorder (with at least minimal verbal ability).	15–25 years	United States	9
3. Delman & Jones [39]	24	Youth who received publicly financed MH services as adolescents. Recruited through flyer advertising with a $25 incentive to organizations frequented by young people.	Semi-structured interviews. Additional Likert scale and “yes” or “no” items. Thematic analysis, phenomenological perspective.	No diagnosis specified.	18–26 years	United States	5
4a. Hovish et al. [24]	11	Young people across six centers who reached the transition boundary between CAMHS and AMHS. Subject to a positive response from the CAMHS or AMHS clinician, young people invited to participate in an interview.	Semi-structured interviews. Parents and professionals also interviewed. Thematic analysis of each case (comprising data from multiple sources, as above).	Diagnoses included: Psychotic disorders, MDD, eating disorder, BAD, chronic suicidal ideation, Asperger’s, anxiety, and OCD.	Not specified.	United Kingdom	6
4b. Singh [33]	Not stated.	Sub-sample of service-users, carers and their care coordinators. Recruitment sources not specified.	Interviews using topic guides. Parents and professionals also interviewed. Analytic method not described.	Diagnosis not specified.	Not specified.	United Kingdom	4
4c. Singh et al. [6]	11	Subsample of service users who had completed transition from CAMHS to AMHS.	Semi- structured interviews. Parents and care-coordinators also interviewed. Constant comparative method.	Diagnosis not specified.	Not specified.	United Kingdom	7
5. Hyde [41]	20	Adolescents in out-of-home placements. Recruitment strategy not described.	Interviews (not described). Professionals who work with foster youth (not necessarily the included youth) also interviewed. Analytic method not described.	No diagnosis specified.	16–18 years.	United States	3
6. Jivanjee & Kruzich [23]	16	Youth referred by MH professionals. Recruitment from local mental health agencies, youth advocacy/support groups, colleges, alternative schools, and youth employment organizations.	Focus groups (not described). Parents also interviewed. Thematic analysis. Constant comparative method.	Diagnoses included: BAD, MDD, LD, ADHD, behavioral disorder, OCD, PTSD.	17–23 years.	United States	8
7. Klodnick et al. [46]	16 (pre-transition) 13 (post-transition)	Purposive sample of young people who planned to exit the therapeutically-oriented transitional living program within one year.	Semi-structured interviews. Grounded Theory, negative case analysis.	Diagnoses included: BAD I, schizophrenia or schizoaffective disorder or MDD.	20.1 years (pre-transition average); 23.1 years (post-transition average)	United States	9
8. Lamont et al. [38]	10	Each local authority asked to identify 4 care leavers willing to act as case studies. Local authorities asked to select young people who had been in care aged 16–21 (or 24 if still in full-time education), and who had mental health needs.	Interviews using topic guides. Professionals also interviewed. Analytic method not described.	Diagnoses included: MDD, suicidal ideation, PTSD, BAD, anxiety, substance use, psychotic disorders, self-esteem issues, behavioral issues.	16–23 years.	United Kingdom	4
9a. Lindgren [37]	3 pre-transition; 11 post-transition	Recruited if between 18 to 25 years old, having experiences of care at both child and adolescent psychiatry (CAP) and General Psychiatry (GenP). Invited to participate by therapist at CAP.	Interview guide with open-ended questions. Family members also interviewed. Grounded theory.	Diagnoses included: Anorexia, anxiety, MDD, suicidal ideation, ADHD, Asperger’s and drug addiction.	18 years (pre-transition); 18–26 years (post-transition)	Sweden	10
9b. Lindgren et al. [36]	3	Participants recruited when terminating care at CAP and referred to GenP. Invited to participate by therapist at CAP.	Interviews (not described). Parents and professionals also interviewed. Grounded theory.	Diagnosis not specified.	Not specified.	Sweden	9
10a. Munson et al. [35]	60	Diagnosed with a mood disorder during childhood, used Medicaid-funded MH services, and used at least one additional public system of care. Recruited through study ads at agencies serving former system youth and in community.	Semi-structured interviews. Additionally, survey Questionnaires: SACA, CESD, CTQ. Immersion/crystallization. Grounded theory.	Mood disorder.	18–25 years.	United States	10
10b. Munson et al. [34]	60	Diagnosed with a mood disorder during childhood, used Medicaid-funded MH services, and used at least one additional public system of care. Participants had to be living in the community.	Semi-structured interviews. Additionally, survey Questionnaires: SACA, CESD, CTQ. Immersion/crystallization. Grounded theory.	Mood disorder.	18–25 years.	United States	10
11. O’Loughlin [45]	6	Youth with eating disorder who have transitioned from CAMHS to AMHS in the past 5 years. Currently receiving treatment for an eating disorder or have undergone a planned discharge from adult services.	Semi-structured interviews. Parents (n = 5) were also interviewed. Interpretative phenomenological analysis.	Eating disorders (5 Anorexia Nervosa and 1 Bulimia Nervosa).	18–21 years.	United Kingdom	10
12. Sakai et al. [43]	28	History of MH service use while in foster care and use of at least one MH service after aging out. Purposive sampling from a community center assisting youth transitioning to adulthood from foster care. Recruited through standardized scripts by on-site case managers.	Semi-structured focus groups. Grounded Theory, coding consensus co-occurrence and comparison method.	No diagnosis specified.	18–27 years.	United States	9
13. Swift et al. [44]	10	Young people aged 17-years and over with a diagnosis of ADHD or psychotic illness. Participants were identified though the young person’s CAMHS clinician.	Semi-structured interviews. Thematic analysis.	Diagnosis of ADHD or psychotic illness.	17 years and over	United Kingdom	8
14. Wheatley et al. [47]	8	All females who had completed a transition from the adolescent medium secure services to the adult female secure services (medium and low secure) within an 18 month period.	Semi-structured interviews. Iterative inductive content analysis.	Diagnoses included: forensic history, emotionally unstable personality disorder, paranoid schizophrenia, post-traumatic stress disorder, attention deficit disorder, substance misuse, self-injurious behavior, history of childhood abuse.	Not specified.	United Kingdom	6

### Quality assessment

Using the CASP tool, we identified issues with quality of included studies (see Table 1 for CASP scores). Overall, many articles scored lower in the areas of methodology. Lower scoring articles were mainly from the grey literature [38–40] or conference abstracts [33, 41] and had limited description of methodology. In other low scoring articles, sampling and recruitment methods typically relied on convenience sampling [23, 34, 35, 39, 42], limited participation to those selected by their service providers [24, 36, 38, 43–45], and/or did not consider the relationship between researchers and participants [6, 23, 24, 33, 36–39, 41, 43, 44, 46, 47].

Other limitations were also identified. Four studies did not operationalize the boundaries of transition from CAMHS to AMHS and the timing of interviews with youth was often unclear [23, 38, 41, 42], making it unclear how much exposure youth had to AMHS. Furthermore, in studies that also examined parent, caregiver or professional views, three studies linked parental and youth views together in the discussion of themes [6, 24, 44]. To keep this thematic analysis in line with views of youth, only findings indicated as originating from youth were used in analysis.

### Thematic foci

Three thematic foci emerged from the thematic synthesis: (1) Complex Interplay of Multiple Concurrent Transitions (2) Balancing Autonomy and the Need for Supports and (3) Factors Impacting Youth Experiences of Transition.

### Complex interplay of multiple concurrent transitions

On the path to becoming an adult, young people experience multiple transitions in a variety of domains in addition to the transition from CAMHS to AMHS, such as change in level of parental involvement, life events, and community agency involvement. For some youth, these transitions were a major obstacle for continued mental health service use, such as described by a youth interviewed by Sakai et al.:

*“I didn’t even get no Medicaid when I left foster care. I had it but, it was never mailed to me. I didn’t even have an address.”* [43], p6.

In contrast, for some, life transitions, such as becoming a parent, were a way for young people to become re-engaged in mental health services as described in Munson et al. [35]:

*“…and shortly after she was born I had talked to the doctor about postpartum depression… I’m like ‘…I don’t want to get out of bed… I don’t want to do nothing’… He was like ‘Well you could be having postpartum depression,’ and that’s when all of my childhood mental problems came up, because he had accessed my information from my old doctor… he’s like ‘Well you know you need to be on this medication…’”* [pg 3].

As youth prepared to undergo major life transitions, some voiced worries about the overlap of these changes with their mental health care transition. The interplay of these transitions made some feel more vulnerable, especially as they sensed the impact of diminishing supports:

*“I turn 18 in like 2 weeks, and I want to move out and live on my own, but it is going to be hard for me because once I turn 18, the supports that I have, some of them are going to disappear.*. *.*. I *am going to have to be able to deal with my issues on my own and find other supports.”* [23, p 11].

Due to these concurrent transitions, young people also expressed a desire for practical support to help them achieve their functional goals as they emerged into adulthood.

*“Leaving the youth system would have been a better experience if someone could have helped me return to school…Somebody helping me reach my goals of getting a university degree”* [39], p19.

*“I wish that we had groups that—when we would make it out in the real world—we wouldn’t be as stunned, you know, dealing with you know the day to day things of paying your rent, paying your cable bill, paying your phone bill...”* [45], p3.

### Balancing autonomy and the need for supports

Many young adults spoke about their perceived lack of knowledge about their own diagnoses, available treatments, and risks or benefits of prescribed medications. One youth described a lack of understanding of the various care provider roles involved in his care:

*“I mean, all that matters to me is just who is it, when is it, where is it … I never care about what their profession is*.” [42], p5.

Youth perspectives around independence and parental involvement varied with the population of the primary study. Youth with eating disorders welcomed the opportunity to receive individually-based care (vs. family-based care) during the transition from CAMHS to AMHS [45]. In contrast, some youth with Autism Spectrum Disorder (ASD) were largely unaware of the transition process, and reported lacking skills and knowledge in how to manage their mental health independently [42], with some questioning the relevance or importance of knowing more [42]:

*“They [medical doctors and mother] said I would never be able to take responsibility for most of the things that happen. So they do it for me … I don’t talk to the doctors at all*.” [p6].

However, other youth with ASD saw the transition from CAMHS to AMHS as an opportunity to gather information about their issues to begin dealing with their aspects of their medical care independently, as described by another youth [42]:

*“I got to start doing it. ‘Cause it’s eventually will be my problem. I’m not just going to call my parents late at night from who knows where I’ll be and ask them about being or going to doctors and wondering where to go…”* [p6].

Though independence was a common theme for many youth, many valued the continued involvement of supports. This was especially the case for youth who experienced gaps in their service provision or decreasing amount of formal supports. For these youth, parents, community agencies and family physicians provided welcomed scaffolding. A youth interviewed by Jivanjee and Kruzich [23] described the important role of his parents:

*“My parents have been a pretty big support, too. I’m 19, so I can’t get support like I did when I was under 18, so my parents had to still kind of stay in there and help me through all the legal troubles and helped guide me through programs, support groups.”* [p11].

### Factors impacting youth experiences of transition

Youth described a number of factors that influenced their experience moving from CAMHS to AMHS (see Table 2 for summary of factors contributing to positive transition experiences). At the pre-transition period, youth emphasized the importance of relationships with staff and positive clinician qualities. In particular, loss of clinician relationships and fear of less support in AMHS characterized youths’ emotional experience, as described by a youth interviewed by O’Loughlin [45]:

**Table 2:** Youth Recommendations for Positive Service Experiences Across the Transition: Pre-, Peri- and Post-Transition Factors

Pre-Transition (CAMHS)	• CAMHS clinician qualities (ex. tenacity, flexibility, instilling hope, providing support and reassurance, non-judgmental, good listener) • Preparation (ex. early notification of transition to AMHS) • Youth involvement in transition planning
Peri-Transition (CAMHS-AMHS)	• Individualized care plans geared towards youth goals of functioning • Increased autonomy in decision-making • Community supports and primary care physicians who provide “scaffolding” across the transition from CAMHS to AMHS • Gradual and flexible timing of transition • Care continuity (ex. “Joint working” or “Parallel Care” between CAMHS and AMHS) • Relational care continuity to reduce fear of losing relationships with pre-transition staff and to promote comfort with AMHS • System-level continuity to reduce gaps
Post-Transition (AMHS)	• Staff support and practical structure • Autonomy in treatment decisions • Choice about parental involvement • Physical care environments geared toward young adults • Informational continuity (ie. sharing of clinical information between CAMHS and AMHS)

“—*I was, was quite nervous and I was quite, I suppose I was quite worried that I wouldn’t get as much support because you always think that children get looked after better than adults, whereas when you’re out in the adult service you wouldn’t get looked after as well.”* [p62].

During the transition, youth felt that the sudden timing of transition was arbitrary, with some questioning the need for a service transition. Young people appreciated transitions that occurred gradually and adapted to their individual needs, in contrast to abrupt, inflexible transition timings.

*“I don’t see what age has got to do with who you ‘re seeing and where you see ‘em. Right, we ‘re used to coming here, but now we ‘ve got to change and go somewhere else, so that’s a bit annoying.”* [44], p6.

*“I think it should maybe be more assessed on the individual patient rather than just ‘oh you’re not 18, so you’re in CAMHS.’ I think that’s maybe something that needs to change…... cause some people are more mature and some people are less mature*.” [45], p61.

*“… gradually, just slowly, slowly I moved up to the adult services when I was ready … I think it was a good transition… I didn’t notice it too much.* ”[24, p4].

Young people wanted to participate in planning the transition collaboratively with care plans that focused on their goals of functioning. Individualized plans based on their unique needs and the opportunity for joint working between CAMHS and AMHS were experienced positively, as described by a youth interviewed by Hovish et al. [24]:

*“I was told about the transfer and I would be meeting the new [AMHS keyworker]…they explained how different it would be…”* [p4].

Once in AMHS, the lack of informational continuity or information sharing between child and adult mental health services was burdensome, and caused youth to have to repeat their personal narrative to multiple clinicians.

“*Even with like therapists that I seen, I seen about four or five, which I always find hard and still do cause you build up trust with somebody and then they disappear and you have to start all over again with the simple questions of when did it start, how did it... and all that”* [45], p77.

Some young adults also expressed difficulty adjusting to the care environment in AMHS:

*“No one understood me. It was awful. I was in the [private] hospital for a while and couldn’t get what I needed. I was one of the youngest people there.”* [39], p17.

*“When I went to CAP I met X regular, but now at GenP it’s like, the worse you feel the more meetings you get, and the better you feel the less meetings you gets, it’s like more irregularity.”* [36], p5.

## Discussion

In this thematic synthesis, we reviewed the experiences of youth transitioning between CAMHS and AMHS. Youth illuminated that the transition was a turbulent time, during which concurrent life transitions overlapped with an institutionalized transition system, that was often unresponsive to individual needs. Youth described a delicate balance between gaining autonomy and independence while also wanting continued parental and service supports. Young people emphasized a need for an individualized approach, that allowed for flexible transition timing, joint working and youth engagement.

Previously, Mulvale et al. [48] described the primary differences in care philosophies between CAMHS and AMHS. Whereas CAMHS emphasized the family unit, AMHS considered youth autonomous adults and limited family involvement in favor of personal privacy. Our review identified that though independence is valued by some youth, for other youth continued parental involvement and support is preferred, and this sudden shift in parental involvement may leave some youth feeling overwhelmed and alone. The abrupt reduction in parental and service-related supports, coupled with the confluence of personal developmental milestones and an institutionalized health care transition creates challenges for youth who are already vulnerable due to mental health difficulties, such as intellectual limitations and emotional vulnerabilities.

Furthermore, there is a shift from a nurturing environment in CAMHS to a more impersonal atmosphere in AMHS [48], which was echoed by young people in our review. Previous research has shown the importance of relationships with clinicians for positive experiences in mental health care [49]. For youth who have previously experienced trauma or family conflict, the loss of important clinician relationships may contribute to poorer transition experiences and overall wellbeing. Difficulty forming new relationships with AMHS clinicians may also lead to a poorer therapeutic alliance and higher risk of disengagement [50].

Youth’s insight into transition timing showed the timing to be arbitrary and misaligned with their own developmental needs. With mental illness frequently emerging in adolescence [1], some youth had only recently become engaged in mental health service use and may have had less comfort and investment with accepting mental health care. Having gaps or suboptimal care during this time may tip these already at-risk individuals into disengagement. Furthermore, current transition timing means transition is rarely an isolated event [3]. Changes in living arrangements, parenthood and other stressors of entering adulthood may take precedence over mental health treatment. Young people’s experiences of inflexible policies, such as the limitations imposed by catchments areas, only add to the difficulty in youth remaining engaged.

Overall, the experiences of youth were closely tied to the distinct cultural differences between CAMHS and AMHS. This is consistent with previous findings by the NHS England, which described the sharp change from child to adult services as a “cliff edge” which can cause a young person to relapse or even stop using services [51]. Health care providers have also shown awareness of this divide and attempts at collaborative working between services [52]. However, these efforts are limited by financial, structural and geographical barriers.

This is the first thematic synthesis specifically aimed at systematically examining and synthesizing the perspectives of youth as they transition from CAMHS to AMHS. Overall, in this study we have synthesized the views of 253 youth. The current review contains four studies [35, 38, 39, 41] that have not been included in previous reviews [18, 22, 25] and provides an opportunity to expand on earlier understandings to a wider population in terms of clinical settings and diagnostic categories. In addition, our methodology allowed us to compare and contrast the experiences of youth across clinical settings and diagnostic categories. Though we identified large overlap in the themes across our included studies with similar issues being important to youth (e.g. parental involvement), our synthesis also shows that the experiences and needs of youth are not homogenous (e.g. desire for continued parental involvement versus desire for independence), which is an insight that would not be obtained from the individual studies alone. Finally, whereas previous reviews have included perspectives of parents and service providers, this review focuses exclusively on the distinct perspective of youth to enable their voices to be heard and to highlight their potential contribution to improved design of services.

### Study limitations

Several limitations of this study should be considered. First, all primary research included in this thematic synthesis was given equal weight, even though there was variability in the quality of the included literature. Due to the reliance on convenience sampling and recruitment through service-providers, service-users included in the studies may have been less likely to have been lost to follow-up and might have had more positive transition experiences. As well, it is possible that included service-users may have been at a higher level of functioning and more able to provide a narrative account of their experiences. Also, a large number of included studies did not explicitly consider the relationship between the researcher and participants, which is important to consider as this is a vulnerable population and may have contributed to social desirability bias.

Furthermore, some diagnostic categories may have been over-represented, such as the large number (*n* = 60) and multiple reports of youth included with primary diagnoses of mood disorders [34, 35]. As identified in this thematic synthesis, this is potentially problematic as youth with different diagnoses may have different perspectives of transition. As well, almost all of the studies originated from the United Kingdom or the United States. However, although these two health care system differ greatly, there was significant overlap of themes across all studies, indicating transferability of youth experiences across health care systems.

Lastly, the combined reporting of parental, service-provider and youth perspectives in some studies made it difficult to discern the youth perspective. This is problematic given that the views of youth and parents often diverged around themes of level of parental involvement [6, 45], independent living skills or managing medical lives independently [7].

### Implications and future research

The findings of this thematic synthesis can inform training and supports for clinicians in both CAMHS and AMHS on how to create more youth-centered transitions, such as implementing flexible transition timing and increasing system-level continuity during the transition (see Table 2 for positive service experience factors).

Furthermore, the field is ready for a tool that would enable broader data collection and a better understanding of youth experiences in diverse contexts. Such an instrument, the Continuity of Care in Children’s Mental Health (C3MH), has been developed to measure continuity of care in children’s mental health but may have limited applicability for transition-aged youth [53], due to the limited age range and small sample size in validation studies to date [*N* = 57 youth ages 14–18]. The findings from this thematic synthesis can be used to generate items specific to exploring the experiences of service-users who are transitioning between CAMHS to AMHS, either for a revision to this survey tool or for a new tool. For example, as shown in our synthesis, youth have individual preferences regarding parental involvement and thus, an item could address the extent to which individual youth’s preference for parental involvement during and after the transition were respected and enabled.

Given the limitations identified above, further efforts to investigate youth experiences and preferences will need to employ recruitment strategies that are able to sample a broader range of service-users (e.g. disengaged youth), to ensure the full diversity of youth perspective is elicited. Future research should also formally operationalize the boundaries of transition of CAMHS to AMHS and be clear about the timing of feedback elicited from youth.

Lastly, future research should engage youth through participatory research methods to harness their unique expertise and insights. Participatory research methods have been used successfully with transition-aged youth using mental health services, with positive effects on youth empowerment [54]. Engaging young people as active co-producers of knowledge also provides the opportunity for young people to identify their priorities for their care [55], which often differ from those of service-providers [56]. Our review identified that young people are insightful about their care and engaging youth in research is an important next step to better understanding and bridging the gap between mental health services and young people’s needs.

## Conclusion

Youth and policy makers have recommended that transitional service models should be youth engaging and peer driven [18–20]. The findings of this qualitative meta-synthesis represent the youth perspective and highlight the importance of an individualized approach that takes into consideration the unique experience and pressures of entering adulthood. Youth have valuable perspectives to guide the intelligent design of services and have an important stake in this process. To ensure that mental health services are truly responsive to youth needs, future research could develop validated tools that measure user experience and continuity of care across the transition from CAMHS to AMHS, and evaluate programs and interventions. Increased youth engagement in research and service design can also help to bridge the gap between CAMHS and AMHS by creating more youth-centered transitions.

## Additional files

Additional file 1: ENTREQ Checklist (DOCX 20 kb)

Additional file 2: Example Literature Search Strategy (DOC 25 kb)

## Abbreviations

AMHS: Adult mental health services
ASD: Autism spectrum disorder
CAMHS: Child and adolescent mental health services
CASP: Critical appraisal skills programme
ENTREQ: Enhancing transparency in reporting the synthesis of qualitative research
