# Supplementary material for: Youth experiences of transition from child mental health services to adult mental health services: a qualitative thematic synthesis
Source: BMC Psychiatry. 2017 Nov 28;17:380. doi: 10.1186/s12888-017-1538-1 (PMC5706294; doi:10.1186/s12888-017-1538-1)
Supplement: Supplementary file 1 — ENTREQ Checklist (DOCX 20 kb) [file 12888_2017_1538_MOESM1_ESM.docx]

**Additional File 1**

**ENTREQ Checklist**

| **No** | **Item** | **Guide and description** | **Response (Page No. in manuscript)** |
| --- | --- | --- | --- |
| **1** | Aim | State the research question the synthesis addresses. | Our primary aim is to provide a more comprehensive understanding of youth experiences of transition from CAMHS to AMHS, through a qualitative thematic synthesis of the extant literature in this area. (Abstract, p.2; Background , p.4) |
| **2** | Synthesis methodology | Identify the synthesis methodology or theoretical framework which underpins the synthesis, and describe the rationale for choice of methodology *(e.g. meta-ethnography, thematic synthesis, critical interpretive synthesis, grounded theory synthesis, realist synthesis, meta-aggregation, meta-study, framework synthesis).* | Thematic Synthesis (Methods p.5) |
| **3** | Approach to searching | Indicate whether the search was pre-planned (*comprehensive search strategies to seek all available studies)* or iterative (*to seek all available concepts until they theoretical saturation is achieved)*. | Pre-planned (See “Search Strategy”, Methods p.6) |
| **4** | Inclusion criteria | Specify the inclusion/exclusion criteria *(e.g. in terms of population, language, year limits, type of publication, study type).* | See “Inclusion and Exclusion Criteria” (Methods, pp. 6-7) |
| **5** | Data sources | Describe the information sources used (e.g. *electronic databases (MEDLINE, EMBASE, CINAHL, psycINFO, Econlit), grey literature databases (digital thesis, policy reports), relevant organisational websites, experts, information specialists, generic web searches (Google Scholar) hand searching, reference lists)* and when the searches conducted; provide the rationale for using the data sources. | See “Search strategy,” (Methods, p.8) |
| **6** | Electronic Search strategy | Describe the literature search *(e.g. provide electronic search strategies with population terms, clinical or health topic terms, experiential or social phenomena related terms, filters for qualitative research, and search limits)*. | See “Search Strategy” for description of search strategy (Methods pp. 5,6) and Additional File 1 for example search strategy |
| **7** | Study screening methods | Describe the process of study screening and sifting *(e.g. title, abstract and full text review, number of independent reviewers who screened studies).* | Title and abstract screening completed in duplicate at abstract level. Full-length screening also completed in duplicate. (See “Data Analysis”, Methods pp.7-8) |
| **8** | Study characteristics | Present the characteristics of the included studies *(e.g. year of publication, country, population, number of participants, data collection, methodology, analysis, research questions).* | Study characteristics presented in Table 1, including sample/setting, methods, diagnoses, age, country (Results p.10) |
| **9** | Study selection results | Identify the number of studies screened and provide reasons for study exclusion *(e,g, for comprehensive searching, provide numbers of studies screened and reasons for exclusion indicated in a figure/flowchart; for iterative searching describe reasons for study exclusion and inclusion based on modifications to the research question and/or contribution to theory development).* | Exclusion was comprehensive. Number of studies excluded at each stage with reasons for exclusion presented in PRISMA chart (See “Figure 1”, Results, p.9) |
| **10** | Rationale for appraisal | Describe the rationale and approach used to appraise the included studies or selected findings *(e.g. assessment of conduct (validity and robustness), assessment of reporting (transparency), assessment of content and utility of the findings).* | CASP tool used for quality assessment of included studies. See “Quality Assessment” for description of rationale and approach (Methods, p.7). |
| **11** | Appraisal items | State the tools, frameworks and criteria used to appraise the studies or selected findings *(e.g. Existing tools: CASP, QARI, COREQ, Mays and Pope* [[25](http://www.ncbi.nlm.nih.gov/pmc/articles/PMC3552766/#B25)]*; reviewer developed tools; describe the domains assessed: research team, study design, data analysis and interpretations, reporting).* | CASP tool (Methods, p.7).  Reference: Critical appraisal skills programme: making sense of evidence. Oxford: Better Value HealthCare. 2002. Available from: http://www.casp-uk.net/. Accessed Feb 27, 2017. |
| **12** | Appraisal process | Indicate whether the appraisal was conducted independently by more than one reviewer and if consensus was required. | Quality appraisal using the CASP tool was conducted in duplicate. Any differences were resolved by consensus amongst the two team members. (Methods p.7) |
| **13** | Appraisal results | Present results of the quality assessment and indicate which articles, if any, were weighted/excluded based on the assessment and give the rationale. | Cumulative CASP scores presented in Table 1 (Results p. 10). All studies were included in final analysis (Methods p. 7). |
| **14** | Data extraction | Indicate which sections of the primary studies were analysed and how were the data extracted from the primary studies? *(e.g. all text under the headings “results /conclusions” were extracted electronically and entered into a computer software).* | Only findings indicated as originating from youth were used in analysis (typically, sections labelled “findings” or “results”) and were extracted into a Word processor (Methods p. 7). |
| **15** | Software | State the computer software used, if any. | DistillerSR, Word, Excel (Methods p.7) |
| **16** | Number of reviewers | Identify who was involved in coding and analysis. | All authors except AC (See “Data Analysis” Methods pp.7-8 for author contributions to coding and analysis). |
| **17** | Coding | Describe the process for coding of data *(e.g. line by line coding to search for concepts).* | See “Data Analysis” (Methods, pp. 7-8 |
| **18** | Study comparison | Describe how were comparisons made within and across studies *(e.g. subsequent studies were coded into pre-existing concepts, and new concepts were created when deemed necessary).* | Analysed three initial studies and generated a coding dictionary. Remaining articles were coded using pre-existing codes, with new codes and revisions to codes made as deemed necessary.  See “Data Analysis”, Methods pp. 7-8) |
| **19** | Derivation of themes | Explain whether the process of deriving the themes or constructs was inductive or deductive. | Inductive, See “Data Analysis” pp. 7-8 |
| **20** | Quotations | Provide quotations from the primary studies to illustrate themes/constructs, and identify whether the quotations were participant quotations of the author’s interpretation. | Original participant quotes used to illustrate themes throughout results section. (Results pp.12-17) |
| **21** | Synthesis output | Present rich, compelling and useful results that go beyond a summary of the primary studies (e.g. *new interpretation, models of evidence, conceptual models, analytical framework, development of a new theory or construct).* | Novel interpretations from primary studies presented through identification and discussion of themes (See Results pp.12-17; Discussion pp.18-22). Recommendations for youth-focused transitions compiled across primary studies in Table 2 (Results, p.17) |
