# Supplementary material for: Youth experiences of transition from child mental health services to adult mental health services: a qualitative thematic synthesis
Source: BMC Psychiatry. 2017 Nov 28;17:380. doi: 10.1186/s12888-017-1538-1 (PMC5706294; doi:10.1186/s12888-017-1538-1)
Supplement: Supplementary file 2 — Example Literature Search Strategy (DOC 25 kb) [file 12888_2017_1538_MOESM2_ESM.doc]

**Additional File 2**

**Example Literature Search Strategy**

**Request: October 13**

**PsychINFO**

1. exp "Continuum of Care"/
2. ((transfer* or transition* or continuity) and (young adult* or youth* or adolescen* or adult*)).ti.
3. ((transfer* or transition* or continuity) adj4 (child* or young adult* or youth* or adolescen* or pediatric or paediatric) adj4 adult*).tw.
4. 1 or 2 or 3
5. exp Adolescent Psychiatry/ or exp Adolescent, Hospitalized/ or exp Adolescent Health Services/ or exp Adolescent Psychology/ or exp Adolescent Development/ or exp Adolescent, Institutionalized/
6. exp Child Psychiatry/
7. youth*.tw.
8. young adult*.tw.
9. adolescen*.tw.
10. child*.tw.
11. 5 or 6 or 7 or 8 or 9 or 10
12. 1 and 11
13. 2 or 3 or 12
14. exp Mental Disorders/
15. exp Mental Health Services/
16. exp Mental Health/
17. exp Psychiatry/
18. exp Drug Abuse/
19. exp Addiction/
20. exp Psychiatric Hospitals/
21. exp Self Injurious Behavior/
22. exp Psychiatric Patients/
23. psychiatry/ or exp community psychiatry/
24. 14 or 15 or 16 or 17 or 18 or 19 or 20 or 21 or 22 or 23
25. 13 and 24
26. 2 or 3
27. 24 and 26
28. 25 or 27
29. Remove duplicates from 28
30. Limit 29 to yr= “1860-2014”

**N= 761**
